# Unpredictable maternal signals and developmental profiles of child executive function from infancy to early childhood

**DOI:** 10.1016/j.dcn.2026.101672

**Published:** 2026-01-09

**Authors:** Fiia Takio, Pilvi Peura, Akie Yada, Anniina Karonen, Pauliina Juntunen, Eeva Holmberg, Eeva Eskola, Elisabeth Nordenswan, Kirby Deater-Deckard, Eeva-Leena Kataja, Asko Tolvanen, Laura Perasto, Elina Mainela-Arnold, Elysia Poggi Davis, Hasse Karlsson, Linnea Karlsson, Saara Nolvi, Riikka Korja

**Affiliations:** a FinnBrain Birth Cohort Study, Turku Brain and Mind Center, Department of Clinical Medicine, University of Turku, Turku, Finland; b Department of Psychology and Speech-Language Pathology, University of Turku, Turku, Finland; c The Centre of Excellence for Learning Dynamics and Intervention Research (InterLearn), University of Turku and University of Jyväskylä, Finland; d Department of Education, University of Jyväskylä, Jyväskylä, Finland; e Helsinki Collegium for Advanced Studies, Finland; f Department of Psychological and Brain Sciences, University of Massachusetts Amherst, USA; g Centre for Population Health Research, Turku University Hospital, University of Turku, Turku, Finland; h Department of Child Psychiatry, University of Turku and Hospital District of Southwest Finland, Turku, Finland; i Department of Psychology, University of Jyväskylä, Jyväskylä, Finland; j Department of Clinical Medicine, Unit of Public Health, University of Turku, Turku, Finland; k Department of Clinical Medicine, Paediatrics and Adolescent Medicine, Turku University Hospital and University of Turku, Turku, Finland; l Psychology Department, University of Denver and the Department of Pediatrics, University of California, Irvine, USA

**Keywords:** Executive function, Early development, Maternal sensory signals, Unpredictability, Longitudinal study, Maternal caregiving behaviour

## Abstract

Early executive function (EF) development is crucial for later cognitive and socioemotional outcomes, yet the role of environmental unpredictability, particularly in patterns of maternal sensory signals, remains underexplored. In this longitudinal study, we investigated the associations between unpredictability in maternal sensory signals and children’s early EF profiles from infancy through the preschool years. Using a population-based birth cohort, we observed a small but significant decrease in the unpredictability of maternal sensory signals over time. This suggests that caregiving predictability may increase as children develop. Nonetheless, within-individual unpredictability showed some stability across time. Importantly, lower unpredictability of maternal sensory signals was associated with membership in more favorable EF profiles, characterized by stronger working memory performance at age five. In contrast, children exposed to more unpredictable maternal sensory signals demonstrated poorer early EF development. These findings build on and extend prior work by modeling unpredictability of maternal sensory signals longitudinally beyond toddlerhood and linking it to children's EF development, highlighting the prolonged sensitivity of EFs to caregiving behavior. Our results underscore that unpredictability in caregiving behavior is a unique and critical factor in shaping early cognitive development and self-regulation. The findings align with emerging cross-species research indicating that patterns of sensory signals are vital not only for sensory processing but also for the development of higher-order cognitive functions. Together, these findings highlight the importance of addressing caregiving unpredictability in early interventions aimed at supporting children’s EF development.

## Highlights

• Unpredictability of maternal sensory signals decreased from infancy to preschool.
• Unpredictable maternal sensory signals associated with poorer executive functions (EF).
• First study to model caregiving unpredictability changes beyond toddlerhood.
• First study linking maternal sensory unpredictability to early EF development.

## 1 Introduction

Unpredictable and stressful environments in early life are well-documented to influence child development (e.g., Davis and Glynn, 2024). However, far less is known about how specific forms of unpredictability—particularly caregiving unpredictability—are associated with fundamental components of psychological functioning. One such important fundament is self-regulation, including the early development of executive functions (EFs). EFs encompass a wide range of processes essential for effortful control and goal-directed behavior (e.g., Diamond, 2013; Miyake et al., 2000). These functions are foundational for later academic achievement, occupational functioning, and socioemotional well-being (e.g., Miller et al., 2012). While EFs are heritable (Friedman et al., 2008, Friedman and Miyake, 2017), early caregiving, parenting styles and caregiving strategies (Bono and Stifter, 2003, Fay-Stammbach et al., 2014, Kochanska et al., 2000, Roskam et al., 2014, Valcan et al., 2018) also influence their development. Investigating specific features of parental caregiving behavior unpredictability is crucial for identifying environmental influences, potential pathways, and mechanisms in early EF development, as well as for distinguishing protective factors from risk factors.

Previous research has shown that early life adversity, particularly in environments marked by unpredictability, can negatively affect the development of EFs in early childhood (e.g. Blair et al., 2011; Ursache et al., 2015). However, research on proximal mechanisms, such as unpredictability in daily caregiving interactions, remains sparse. One key reason is the challenge of conceptualizing and reliably measuring unpredictability at the level of caregiver behavior (Baram et al., 2012, Davis et al., 2019, Davis et al., 2017). A novel, translational approach addresses these gaps in the previous literature by quantifying the unpredictability of maternal sensory signals during interactions with the child (Baram et al., 2012, Davis et al., 2022, Davis and Glynn, 2024). This proximal level of unpredictability in caregiving is captured by the entropy rate, an index of the unpredictability in transitions between maternal sensory inputs (e.g. visual, auditory, and tactile). Higher entropy rate reflects more inconsistent patterns of sensory signalling (Davis et al., 2022). Although maternal entropy is not conceptualized as early life adversity in the same sense as severe experiences such as maltreatment, it is considered to reflect variation within normative caregiving environments that may nonetheless influence the development of stress regulation systems. The early life adversity framework is applied here to emphasize that such unpredictability may affect neurodevelopment through mechanisms similar to those implicated in more traditionally defined adversities (Baram et al., 2012, Davis et al., 2017). Importantly, the entropy rate has shown to be distinct from traditional maternal sensitivity metrics, with each demonstrating independent associations with child development (Holmberg et al., 2022a, Holmberg et al., 2022b).

Emerging evidence associates higher unpredictability in maternal sensory signals with poorer child socioemotional development (Aran et al., 2024), impaired stress regulation (Noroña-Zhou et al., 2020) and neurocognitive challenges including reduced working memory and attention regulation (Davis et al., 2017) as well as effortful control (Davis et al., 2019; Holmberg et al., 2022b), constructs closely related to EF (Chae, 2022, Best and Miller, 2010, Rothbart and Bates, 2007). However, to date, no studies have directly examined how maternal sensory signal unpredictability relates to the development of EF across early childhood. This gap is notable given young children’s reliance on caregivers for stimulation, care, and for regulating their emotions and behavior (Sameroff, 2010), as well as the sensitivity of EF-related brain circuits to early environmental inputs. It is plausible that patterns of maternal sensory signals influence the organization of EF-related neurocognitive circuitry, especially since early experiences play a crucial role in shaping the development of prefrontal cortex (PFC) circuitry in the brain, closely tied to EF development and characterized by high neural plasticity during early childhood (Fiske and Holmboe, 2019, Kolb et al., 2012). Recent mechanistic research demonstrates that early unpredictability of maternal sensory signals impacts neurodevelopment by altering hippocampal–prefrontal connectivity and stress-related pathways. This occurs through disruption of critical processes such as synaptic plasticity (including synaptic pruning and reduced dendritic complexity), circuit formation, and stress hormone regulation (Birnie and Baram, 2022, Davis et al., 2017). These neurobiological systems, particularly the hippocampus and prefrontal cortex, are foundational for EFs such as attention regulation, working memory, and inhibitory control. Their coordinated development begins in infancy and toddlerhood, and is thought to be shaped by early caregiving experiences, including the temporal predictability of sensory input (Gee and Cohodes, 2021, Glynn and Baram, 2019, Herrero-Roldán and Martín-Rodríguez, 2025, Lee et al., 2017). Despite these insights, longitudinal research on maternal caregiving behavior and its relation with child’s early EF development remains surprisingly limited (Wei et al., 2023). In the present study, we aim to investigate both the change and stability in the unpredictability of maternal sensory signals throughout early childhood, and how these patterns are associated with the longitudinal development of EFs from infancy to the preschool years.

### 1.1 Parental caregiving behavior influences early development of child executive function skills

Although the construct of early EF remains a topic of debate (e.g. Sambol et al., 2023), it is generally regarded as a multi-component construct comprising a set of dissociable yet interrelated cognitive processes. Core elements of EF —working memory (WM), inhibitory control (IC), and cognitive flexibility (FX) — are foundational for adapting to environmental changes and goal-directed behavior. WM involves maintaining and updating information in short-term memory, IC refers to ability to suppress dominant, automatic, or prepotent responses stimuli, and cognitive flexibility (i.e. set shifting) indicates to the ability to adaptively shift attention between tasks, strategies, or mental sets (Cristofori et al., 2019). Together with attentional control these core functions form the foundation for more complex EF skills, such as planning, problem-solving and organizing (Diamond, 2013, Miyake and Friedman, 2012).

Research on the early development of EFs is mixed, in part due to the concurrent development of various other cognitive abilities like language, along with the evolving structure of EFs, together complicating the measurement and modeling of very early EFs (Ribner and Holmboe, 2024). Additionally, the core components of EFs do not appear to be uniform constructs; rather, they represent latent factors composed of distinct dimensions and subsystems, each with different developmental onsets and marked interindividual variability (for a review, see Best and Miller, 2010; see also Friedman and Miyake, 2017; Gandolfi et al., 2014). EF skills begin to emerge in infancy, with marked growth between the ages 3 and 5—when children are particularly reliant on caregivers (Diamond, 2013, Edgar et al., 2023, Hendry et al., 2016; Holmboe et al., 2018; Kolb et al., 2012; Morgan et al., 2025). While some longitudinal research suggest that the stability of individual differences in EFs can be detected as early as two years of age, the variability of results highlight the need for further investigation (Broomell and Bell, 2022, Garon et al., 2008, Miller et al., 2023). Measuring early EF skills with tasks longitudinally often introduces challenges, as the difficulty level of tasks must align with the maturation of the child's cognitive and motor abilities. This alignment can increase measurement error, as the tasks administered may not consistently assess the same aspects of EF across different ages. To address these key methodological challenges, such as modeling the longitudinal development of EF using developmentally appropriate measures and managing the task impurity problem in EF assessment (Friedman and Miyake, 2017), we build upon our two recently completed studies: one applying item response theory (IRT) to enhance measurement precision (Yada et al., 2025) and another using IRT to model early EF development with latent profile analysis (LPA) to reliably identify longitudinal EF developmental profiles during early childhood from infancy to preschool years (Karonen et al., 2025).

The current body of literature demonstrated that early development of EF is dependent on the interplay of multiple individual-level factors and environmental factors. One set of factors is related to early caregiver characteristics which are known to be pivotal for early childhood development. For instance, the quantity and quality (i.e. sensitivity, scaffolding, stimulation or behavioral control vs. harshness, insensitivity, intrusiveness or hostility) of parental care in infancy associates to children’s later emotional well-being, cognitive development (e.g. EF) and social functioning (Fay-Stammbach et al., 2014, Lucassen et al., 2015). For example, it is suggested that there are critical or sensitive periods when environment has stronger influences on the development of child’s EF (see Thompson and Steinbeis, 2020 for a review). However, the exact timing of these periods remain under debate (e.g. Hendry et al., 2016). A meta-analysis by Valcan et al. (2018) indicated that socioemotional and cognitive parenting practices — including warmth, control, and cognitive stimulation — relate to children’s EFs, both globally and in specific domains like IC, WM and FX. Notably, the link between cognitive parenting and global EF weakened as children aged. Roskam et al., (2014) reported that variation in parenting style influences IC development in children aged 2–8 years of age. Similarly, Bono and Stifter (2003) observed that children whose mothers used attention-maintenance strategies during play showed better attention and cognitive function at 18 months than those whose mothers used redirecting strategies.

In summary, each component of EFs appears to develop at its own pace, beginning in childhood and continuing through later stages of life, with different aspects maturing at various ages. EF skills continue to evolve throughout adolescence and beyond (e.g. Ferguson et al., 2021; Takio et al., 2014). Early childhood may be a critical period during which parental behaviors significantly influence the development of a child's later EF skills. This underscores the importance of understanding the role of the early parenting environment, particularly the specific caregiving factors and underlying mechanisms that contribute to unpredictability in the child’s environment and may be of relevance for EF development.

### 1.2 Exposure to unpredictability of maternal sensory signals in caregiving behavior during early development

Emerging brain circuits are shaped during sensitive or critical developmental periods in early life during which there is heightened neural sensitivity to specific environmental stimuli, with exposure necessary for typical developmental processes to occur (e.g. Birnie and Baram, 2022; Davis and Glynn, 2024; Luby et al., 2020; Thompson and Steinbeis, 2020; Tottenham, 2013). Environmental input plays a crucial role in this process, as repeated activation strengthens synaptic connections, while those used less frequently are pruned. Patterns of sensory information have shown to shape the development and potentiation of brain synapses and circuits in several sensory domains, and thus regulate and modify the child’s neurodevelopment and higher order cognitive functions (for review, see Davis et al., 2022; see also Birnie and Baram, 2022; Luby et al., 2020). It has been suggested that the unbalanced maturation of corticolimbic circuits is a mechanism through which early unpredictable sensory signals may affect higher cognitive processing later in life (Birnie and Baram, 2022, Granger et al., 2021). Specifically, early unpredictability may influence the development of hippocampal-prefrontal connectivity and stress-related pathways, which are foundational for EFs such as attention regulation, working memory, and inhibitory control. These neural systems begin to organize during infancy and toddlerhood, and their coordinated maturation is thought to support emerging EF skills (Lee et al., 2017, Gee and Cohodes, 2021, Herrero-Roldán and Martín-Rodríguez, 2025). In addition, early development of intersensory processing skills, which require the ability to detect and selectively attend to synchronously co-occurring intersensory redundancy, is also thought to be a foundation for later developing cognitive functions such as language, EF, and social functioning (e.g., Edgar et al., 2023).

A set of a recent studies has demonstrated that exposure to unpredictable maternal sensory signals early in life predicted poorer cognitive development later in life across species (Hyysalo et al., 2024, Davis et al., 2017, Davis et al., 2019). More specifically, less predictable maternal sensory signals at the age of 1 year predicted poorer cognitive performance at the age of 2 years, and this association persisted when the child was 6.5 years old (Davis et al., 2017). In a cross-cultural design conducted in Finnish and US samples of infants, unpredictable maternal sensory signals were associated with low effortful control across infancy (Davis et al., 2019). Moreover, the association between unpredictability and effortful control persisted until 9.5 years of age in the US sample (Davis et al., 2019) and until 2 and 5 years in the Finnish sample (Davis et al., 2019; Holmberg, et al., 2022b). Finally, early exposure of predictable caregivers’ auditory sensory signals in infancy was associated with learning-related changes in infant’s neural responses to predictable auditory information during statistical learning task approximately five months later (Forest et al., 2025). These findings together indicate that unpredictability of maternal sensory signals indeed has relevance for children’s cognitive development in the long-term. However, to our knowledge, all these previous findings have focused on the effects of unpredictable maternal signals during infancy and their impact on later child development. While some caregiving behaviors, such as maternal sensitivity, appear to show moderate individual stability throughout infancy and toddlerhood (e.g. Bigelow et al., 2010; Dallaire and Weinraub, 2005; Holmberg et al., 2022a), this may not be true for all caregiving behaviors. In contrast, a more recent study using a subset of the current cohort found that the unpredictability of maternal sensory signals showed a modest decrease over time, despite relatively stable individual differences (Holmberg et al., 2022a). However, we are not aware of prior studies that has investigated the stability versus change in the predictability of maternal sensory signals during mother-child interactions beyond toddlerhood, nor the impact of long-term exposure to unpredictable maternal sensory signals on children's neurocognitive development.

Taken together, the findings on sensory input and higher-order cognitive development highlights the importance of investigating the association between patterns of unpredictable maternal sensory signals and child EF development. Given that EF development is closely linked to the prolonged maturation of the PFC (Fiske and Holmboe, 2019), exploring the effects of longitudinal exposure of maternal caregiving behavior on child’s EF development beyond infancy appears particularly relevant, especially when designing parenting interventions aimed at supporting child’s early EF development. In response to this identified research need, the present study aims to longitudinally investigate the associations between the unpredictability of maternal sensory signals during mother-infant interaction, characterized as the entropy rate, and children’s early EF developmental profiles from infancy to preschool years in the FinnBrain Birth Cohort Study.

The first aim of the study was to examine whether the unpredictability of maternal sensory signals, quantified as entropy rate, changes over time when measured at three time points: 8 months, 2.5 years, and 5 years. A previous study from our research team, using a subsample of the current cohort, found a decrease in entropy rate from 8 months to 2.5 years (Holmberg et al., 2022a). However, as no prior research has investigated the persistence of entropy rate beyond toddlerhood or in other samples, we did not formulate specific hypotheses regarding its trajectory across early childhood.

The second aim was to investigate whether the unpredictability of maternal sensory signals during mother-child interactions, measured at the same three time points (8 months, 2.5 years, and 5 years), is associated with the child’s developmental executive function (EF) profiles (Karonen et al., 2025). Prior research has linked unpredictable maternal sensory signals in infancy to poorer neurocognitive outcomes later in life (e.g., Davis et al., 2017, Davis et al., 2019). Moreover, emerging evidence suggests that unpredictable environments may shape, rather than uniformly impair, cognitive functions related to EF (e.g., (Young et al., 2018). Based on this, we hypothesized that entropy rate during early childhood would be associated with the development of EF, although the direction of this association remains unclear.

## 2 Materials and methods

### 2.1 Participants

The participants in the present study were a subsample (N = 838, 99.9 % White) of children from the population-based FinnBrain Birth Cohort Study (N = 3808 families) (Karlsson et al., 2018). The participant families recruited and enrolled provided written informed consent in their first trimester (gestation week 12) ultrasound visit between December 2011 and April 2015. FinnBrain Focus cohort sample included children whose mothers experienced either low, moderate, or high prenatal distress. Prenatal psychological distress in the FinnBrain Focus Cohort was assessed using repeated measures of depressive symptoms (EPDS), general anxiety (SCL-90), and pregnancy-specific anxiety (PRAQ-R) at gestational weeks 14, 24, and 34. For the selection of the focus cohort, prenatal psychological distress was defined as elevated maternal symptoms on these measures. Case classification was based on two positive screenings above predefined cut-off points—either twice using the same instrument or once using two different instruments—and included SSRI use. This resulted in approximately 20 % cases and 27 % controls out of the full cohort of 3808 mothers. The selected cut-off points, based on quartile distributions, were as follows: EPDS > 11 points (maximum score: 30) for depression; SCL-90 General Anxiety Scale > 9 points (maximum score: 40) for general anxiety; and Pregnancy-Related Anxiety Questionnaire Revised > 33 points (maximum score: 50) for pregnancy-specific anxiety. Control participants scored within the lowest quartile on all questionnaires at each measurement point during pregnancy: EPDS < 7 points, SCL-90 < 5 points, and PRAQ-R < 26 points. (for more details, see Karlsson et al., 2018). The Focus Cohort was then enriched in subsequent age points by other actively participating cohort families so that the sample represents baseline population of the cohort. The subsample of the present study consists of 838 families that took part in the 8- month, 30- month and/or 5 years neuropsychological assessment visit and thus had available data for the modeling of EF profiles (Karonen et al., 2025) and/or had available data for mother-child interaction measurements at the FinnBrain Birth Cohort Research site. More precisely, 830 children had EF data enabling EF latent profile analysis, 659 children had mother-child interaction data to enabling latent growth curve analysis for the entropy rate, and 651 children had both EF and mother-child interaction data. The 8 months data was collected between June 2013 and June 2016, the 2.5 years data between April 2015 and June 2018 and the 5 years data between October 2017 and December 2020. The demographic details, the number of participants from the different study visits and the background variables included in the present study are represented in more detailed in the Table 1.

**Table 1:** Demographic characteristics.

Characteristic	Range	Mean (SD)	No. (%)
**Child Characteristics**			
Child sex assigned at birth (%)			
male			466 (55.6)
female			372 (44.4)
Child age (months)			
At 8-month visit	6–9	7.6 (0.54)	
At 2.5-year visit	28–32	29.6 (0.57)	
At 5-year visit	58–64	59.7 (0.91)	
Duration of gestation (weeks)	29.3–42.4	39.76 (1.66)	
Children born before 37 weeks of gestation			47 (5.7)
**Maternal Characteristics**			
Maternal age at delivery	18–45	31.0 (4.54)	
Financial satisfaction, early 2nd trimester (0−10)	0–10	5.91 (2.40)	
Financial satisfaction at child age of 5 y (0−15)	1–15	9.95 (3.29)	
Average financial satisfaction (standardized)*	-2.46–1.70	0.04 (0.91)	
Maternal education **			
High school/vocational education or lower			201 (24.2)
Vocational tertiary/applied university			250 (30.1)
University degree			368 (44.3)
Maternal depressive symptoms ***			
At 6-months		4.42 (4.32)	
At 24-months		4.52 (4.30)	
At 5-year		4.82 (4.57)	

** Most recent/highest known information on maternal education

*** Measured with Edinburgh Postnatal Depression Scale (EPDS)

* Standardized mean of the two time points (gw 14 and 5 years)

### 2.2 Procedures

The data in all age points were carried out during individual 90 min (8 months and 2.5 years) and two-hour (5 years) laboratory visits at the FinnBrain Child Development and Parental Functioning lab. The questionnaires, neuropsychological assessments and mother-child play sessions were conducted by the clinical psychologists or trained psychology master’s students. The Ethics Committee of the Hospital District of Southwest Finland approved the study protocol (ETMK: 57/180/2011; ETMK: 59/1801/2013; ETMK 103/1801/2014). Sociodemographic information affecting early neurocognitive development was obtained either by wellbeing services county of Southwest Finland (VARHA) records (age when tested (days), sex (1 = females; 2 = males), gestation age (weeks)) or by maternal self-report (level of education (gestational week (gw) 14), maternal financial satisfaction (gw 14 and 5 years of age), depressive symptoms (6 month, 2.5 years, and 5 years).

### 2.3 Measures (Key variables)

#### 2.3.1 Measuring the degree of unpredictability of maternal sensory signals i.e. entropy rate

Mother–child interaction was assessed three times (at 8 months, 2.5 years and 5 years) in a video-recorded 10- min free-play play episodes. The mother was offered a standard set of toys and asked to play with her child for 20 min, as they normally would at home. The first ten minutes were selected for coding maternal sensory signals.

##### 2.3.1.1 Coding maternal sensory signals

The maternal sensory signals (i.e., auditory, tactile, visual) were coded from 10 min sequences of mother-child play episodes continuously at momenton-moment basis using The Observer XT 11 (Noldus). The maternal sensory signals were coded by trained double-blinded coders from the video recordings. Inter-rater agreement was calculated for 10 % of the videotapes and was 86 % at 8-months measurement, 84 % at 30-months measurement and 84 % at 5 years measurement. Tactile signals included all physical contact between the mother and child (e.g., touching, holding). Auditory signals encompassed all maternal vocalizations (e.g., laughing, vocal sounds, talking). Visual signals referred to all instances where the mother manipulated a toy or object while the child was visually attending. (Davis et al., 2017).

##### 2.3.1.2 Quantifying unpredictability of maternal sensory signals i.e. entropy rate

In the coding approach, maternal sensory signals were coded separately for visual, auditory and tactile sensory signals (Davis et al., 2017). For example, the auditory and visual input was coded when the mother simultaneously spoke to her child while showing a toy to her/him. If she then additionally touched the child, all three sensory inputs (auditory, visual and tactile) were provided simultaneously, and this would be considered a transition to a different combination of sensory signals provided by mother. Transitions between different combinations of maternal sensory signals (eight possible combinations based on the presence or absence of each of the three types of sensory input) were modeled as changes in the state of a discrete-state Markov process (Vegetabile et al., 2019). The entropy rate of this process was then used as a measure of the unpredictability of maternal sensory signals. Highly predictable maternal sensory signals indicate that one or combination of signals always followed another (e.g. touch always followed by speech and visual input), the maternal signals would be considered highly predictable for the child indicating a low entropy rate. In turn, if the probability of one maternal signal following another was random, the maternal signals would be considered highly unpredictable, indicating a high entropy rate. The entropy rate can range from 0 to 3, higher values indicating more unpredictable maternal sensory signals and caregiving behavior. For more detailed information and materials, see Davis et al. (2017) and https://contecenter.uci.edu/shared-resources/.

#### 2.3.2 Longitudinal profiles of executive functions from infancy to 5 years of age

**Fig. 1:**
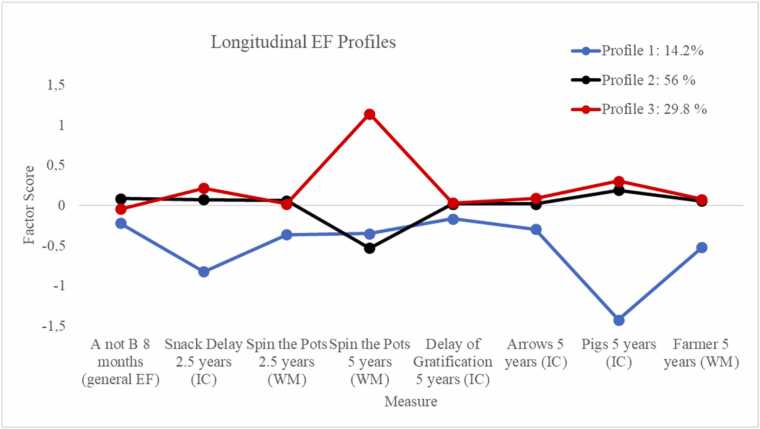
The profiles of EFs across tasks N = 830, (Karonen et al., 2025). Profile 1 = Below average performers, Profile 2 = Average performers with lower Spin the Pots (5 y) performance, Profile 3 = Average performers with higher Spin the Pots (5 y) performance.

For the longitudinal modeling of EFs from infancy to age 5 (Karonen et al., 2025), a combination of age-appropriate, laboratory-based tasks conducted at 8 months, 2.5 years, and 5 years were used, as described in more detail in Supplement. Following the analysis published in our previous research in the same sample (Karonen et al., 2025), children’s longitudinal EF profiles were identified with latent profile analysis (LPA) based on EF task estimates. Factor scores of EF tasks were calculated using item response theory (IRT), which improves measurement precision by accounting for item-level difficulty and discrimination across tasks with varying psychometric properties (for a detailed description of the IRT analysis for each task see Yada et al., 2025). Due to the developmental nature of EF and the use of different age-appropriate tasks at each time point, traditional longitudinal modeling approaches such as latent growth modeling were not applicable, as they require consistent measurement across time. In contrast, LPA was better suited for this context, as it allows for the identification of meaningful subgroups based on EF task performance across heterogeneous measures. LPA was therefore selected as a person-centered approach to identify distinct EF profiles across time points. The focus of this study was on profile membership and its associations with predictability of maternal sensory signals, not on modeling individual developmental trajectories or change processes. The 3-profile solution fitted the data best (Karonen et al., 2025). The fit statistics for LPA for 1 through 5 class solutions is presented in the Supplement (Table S1) and in more detail in (Karonen et al., 2025). The three profiles are graphically illustrated in Fig. 1. In the first profile, labelled as “*Below average performers*” (14.2 %), the children performed below average in the EF tasks in all the time points. The two other profiles were highly similar, except their performance in the Spin the Pots task and were thus labelled “*the Average performers with lower Spin the Pots (5 y) performance*” (56.0 %) and “*the Average performers with higher Spin the Pots (5 y) performance*” (29.8 %). Male sex assigned at birth and lower general cognitive performance at age five were significantly associated with an increased likelihood of membership in the *'Below average performers'* group.

#### 2.3.3 Covariates

We included several covariates previously linked to maternal caregiving behavior to control for background factors, specifically indicators of socioeconomic status and maternal depressive symptoms (e.g. Goodman et al., 2020; Holmberg et al., 2022a) and child’s sex assinged at birh (Holmberg, et al., 2022a; Morawska, 2020). Regarding SES, level of maternal education was formulated as a three-group variable (1 = low: secondary education or lower, 2 = middle: tertiary vocational education, 3 = high: university degree) by using the highest education known when the child was 5 years of age. Maternal economic satisfaction (i.e., economic needs were begin met) was asked on a continuous scale from 1 to 10 during pregnancy at gestation week 14, or from 1 to 15 at child age of 5 year, and standardized mean of these two time points were used. Maternal age when their child was born (years), child biological sex assigned at birth (1 = boy, 2 = girl) and length of gestation were drawn from the wellbeing services county of Southwest Finland (VARHA) records. Depressive symptoms were based on mothers self-reported psychological distress (Edinburgh Postnatal Depression Scale (EPDS) (Cox et al., 1987) were obtained at the child age of 6 month, 2.5 years, and 5 years. (see Table 1). In addition, maternal sensitivity was included as a covariate for the sensitivity analysis and was assessed at 8 months, 30 months, and 5 years using the Emotional Availability Scales (Biringen, 2008; scale range 1–7, with higher scores indicating greater maternal sensitivity). A mean score across these three time points was used in the analysis.

### 2.4 Statistical analyses

Descriptive statistics were depicted with SPSS software (version 28). The main analyses were performed using the Mplus software (version 8.11). There was some missing data for the unpredictability of maternal sensory signals, particularly at the 8-months timepoint (5 years: 52.3 %, 2.5 years: 54.9 % and 8 months: 78.5 %). However, the missingness was due to study design*;* The collection of data on mother–infant interactions was added later to the protocol of the FinnBrain Child Development and Parental Functioning Lab study visit at 8 months, which resulted in a smaller subsample compared to the full study visit. For the mother–infant interaction assessments, 354 families were contacted and invited to participate in the study visit when the child was 8 months old. Of those invited, 197 families (55.7 %) participated in the mother–child interaction evaluation. Little’s (1988) missing completely at random (MCAR) test showed that data were not missing completely at random (χ2(3961) = 4384.48, p < .001). Missingness was handled by using The Full Information Maximum Likelihood (FIML) method and 500 starting values.

Longitudinal modeling of the unpredictability of the maternal sensory signals (the entropy rate) and its correlates over the time points of 8 months, 2.5 years, and 5 years were examined with latent growth curve modeling (LGM; Muthen and Khoo, 1998). To find the best fitting model, both linear and nonlinear shapes of change were examined. The following potential variables for the further covariate analysis were examined: maternal age when child was born (years), maternal depressive symptoms, maternal education level, maternal financial satisfaction and child’s sex (see Table 1). Those covariates that were significantly associated with entropy rate were included as a covariate in the model by allowing them to influence latent growth factors (i.e., intercept and slope). To identify the optimal LGM model for the data, *χ2* test was used as well as the values of comparative fit index (CFI, Bentler, 1990), Tucker–Lewis index (TLI, Tucker and Lewis, 1973), root mean square error of approximation (RMSEA, Steiger, 1990) with a 90 % confidence interval, and standardized root mean square residual (SRMR, Hu and Bentler, 1995) were considered. For CFI and TLI values higher than 0.95 and for RMSEA values smaller than 0.06 and for the SRMR values smaller than 0.08, the model was considered to be a well-fitting representation of the data (Hu and Bentler, 1999). To examine whether the level and possible rate of change in the unpredictability of the maternal sensory signals relates to longitudinal EF profiles the Bolck–Croon–Hagenaars (BCH) approach (Asparouhov and Muthén, 2018; Bakk and Vermunt, 2016) was used. The BCH procedure estimates a weighted multiple group analysis to examine the differences between the longitudinal EF profiles as a function of the level and change in the unpredictability of maternal sensory signals. BCH procedure considers the measurement error related to the classification of children into the longitudinal EF profiles using weights that are inversely related to the classification error probabilities obtained from the LPA (Bakk et al., 2013). The manual BCH method was used (Asparouhov and Muthén, 2018) to examine differences in latent variables (i.e., the level and slope of unpredictability in the maternal sensory signals) obtained from the LGM across EF profiles. We included covariates that were significantly associated with entropy rate in the analyses. The overall differences in the means of the level and change in the unpredictability of the maternal sensory signals between the longitudinal EF profiles were examined using likelihood ratio tests. To examine pairwise comparisons between the profiles, Wald tests were used and effect size was measured using Cohen’s d (Cohen, 1988). In addition, as a sensitivity analysis, we included maternal sensitivity as a covariate in a separate model to control for its potential effect on the association between longitudinal EF profiles and maternal sensory signals.

## 3 Results

The descriptive statistics and the bivariate correlations for the studied main variables are presented in the Appendix A (Tables A.1 and A.2).

### 3.1 Longitudinal modeling of the unpredictability of the maternal sensory signals (entropy rate)

**Fig. 2:**
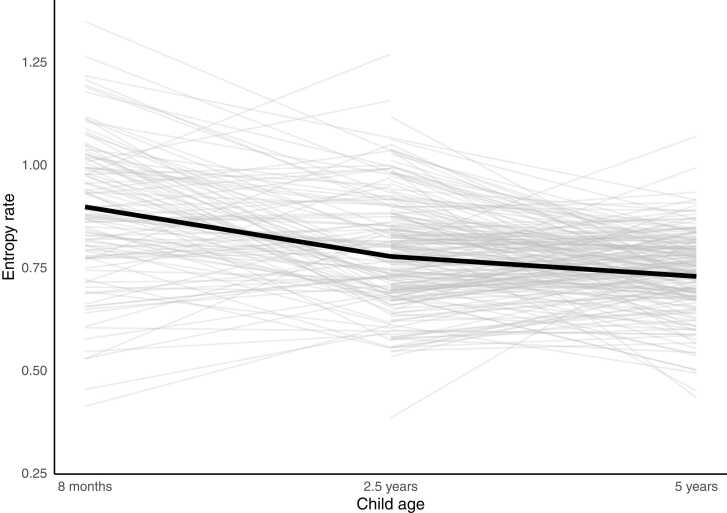
Individual and overall mean change overtime in the entropy rate from child’s age 8 months to 5 years.

First, we investigated changes in entropy rate over the three time points of 8 months, 30 months and 60 months with LGM. The entropy rate measured at 8 months, 2.5 years and 5 years correlated significantly over the time points (r = .25–.33). Of the potential covariates considered, only maternal age and maternal education correlated significantly with the entropy rate (see Appendix A, Appendix B) and were included as covariates in the model investigated with LGM by allowing them to influence latent growth factors (i.e., intercept and slope). The models including the change factor did not fit the data well. There was a significant negative change (estimate –0.11, see Fig. 2) in the entropy rate but no variance in the slope factor indicating that the maternal sensory signals changed towards more predictable, and the rate of change did not differ significantly between the participants. The best fitting model for the data was a model including the level factor of the entropy rate (*χ2*(6) = 6.44, *p* = .376; RMSEA = 0.01; CFI = 0.99; TLI = 0.99; SRMR = 0.07). Maternal age and educational level were negatively related to the level of entropy rate, that is, to the intercept of entropy (*β* = −.21, *β* = −.19, respectively). That is, the younger the mother and the less educated, the more unpredictable the maternal sensory signals were. Maternal economic satisfaction (*β* = −.07, p = 0.29) and child sex (*β* = −.10, p = 0.15) were not significantly related to the level factor and were thus not included as a covariate in further analysis.

### 3.2 Associations between the unpredictability of maternal sensory signals (entropy rate) and child’s longitudinal EF profiles

Analyzed with BCH method, the Wald test was significant indicating that entropy rate differed between the three EF profiles (Wald *χ2* (2) = 7.24, *p* < .05). That is, unpredictability of maternal sensory signals was related to the child's longitudinal EF profiles. Maternal age and educational level were covaried from the entropy level in line with the LGM modeling (*β* = −.015, *β* = −.003, respectively). The results of the Wald tests and the effect sizes reflecting the differences between the estimated level of entropy rate between the EF profiles are presented in Table 2.

**Table 2:** Effect sizes (Cohen’s d) based on pairwise comparisons of the estimated level of the unpredictability of maternal sensory signals by EF profiles.

Profile Comparisons	Wald *χ2* test	Cohen’s d
**1 vs 2** (*Average performers with lower Spin the Pots (5 y) performance***vs***Below average performers*)	0.00	0.00
**1 vs 3** (*Average performers with lower Spin the Pots (5 y)***vs***Average performers with higher Spin the Pots (5 y) performance*)	4.44*	0.55
**2 vs 3** (*Below average performers***vs***Average performers with higher Spin the Pots (5 y) performance*)	5.08*	0.68

*Note*. Effect sizes ranging from 0.10 to 0.30 are considered as small effects, 0.30–0.50, as intermediate effects, and 0.50 and higher, as strong effects (Cohen, 1988). * *p* < .05

The pairwise comparisons between the profiles showed that “*the Average performers with higher Spin the Pots (5 y) performance*” (entropy mean = 0.90) profile differed significantly from the two other EF profiles: “*the Average performers with lower Spin the Pots (5 y) performance*” (entropy mean = 0.94) and “*the Below average performers*” (entropy mean = 0.94) (*ps* <.05). In other words, lower unpredictability of maternal sensory signals was associated with the child’s better EFs during the first 5 years of life. Effect sizes of the comparisons were strong (Cohen, 1988). “*The Average performers with lower Spin the Pots (5 y) performance*” profile and “*the Below average performers*” profile did not differ from each other in the unpredictability of maternal sensory signals. In addition, after covarying the mean score of maternal sensitivity across three measurement points as a sensitivity analysis, the results remained consistent. Post hoc comparisons indicated significant differences in entropy between the following profiles: (1) “*the Average performers with higher Spin the Pots (5 y) performance*” vs. “*the Below average performers*” (estimate = 0.034, *p* < .05) and (2) “*the Average performers with higher Spin the Pots (5 y) performance*” vs. “*the Average performers with lower Spin the Pots (5 y) performance*” (estimate = 0.033, *p* < .05). No difference was observed between the two lower-performing profiles (*p* = .95).

## 4 Discussion

The primary aim of this study was to longitudinally examine the associations between the unpredictability of maternal sensory signals during mother-infant interactions, characterized as entropy rate, and children’s longitudinal latent profiles of early EF development from infancy through the preschool years. To this end, we employed repeated assessments of maternal sensory signal unpredictability, modeling their longitudinal associations with children's early EF development.

First, the stability of unpredictability in maternal sensory signals was examined from infancy to toddlerhood and into the preschool years. Our results revealed a small but significant decrease in unpredictability of maternal sensory signals across this developmental period in our population-based birth cohort sample. To our knowledge, this is the first study to longitudinally model potential changes in the unpredictability of maternal sensory signals beyond toddlerhood as the child develops. While other caregiving behaviors, such as maternal sensitivity, are often considered relatively stable individual characteristics (e.g.,Bigelow et al., 2010; Dallaire and Weinraub, 2005; Holmberg et al., 2022a), the present study similarly found that the unpredictability of maternal sensory signals (entropy rate) exhibited some stability over time, as indicated by significant correlations across time points. However, latent growth model analyses revealed a small but significant decrease in unpredictability of maternal sensory signal over the first five years of life, suggesting that maternal sensory signals became slightly more predictable as children grew older. Notably, this developmental change did not vary significantly between mothers. Given the small magnitude of this change, it is plausible that the unpredictability of maternal sensory signals reflects a somewhat stable aspect of caregiving behavior, albeit one that can show modest developmental change. This shift may indicate that as children develop, mothers acquire better parenting skills, thereby enhancing caregiving predictability. Additionally, the influence of children's cognitive and socioemotional development on mother-child interactions may contribute to increase predictability over time. Together these factors highlight the need for further investigation over longer follow-up periods to better understand the longitudinal changes and the nature of the predictability of maternal sensory signals and the underlying mechanisms. Future studies should examine how age-related changes in the frequency and diversity of maternal behaviors may influence entropy scores, to better distinguish developmental shifts in behavior from changes in temporal unpredictability. This is particularly relevant given that certain behaviors, such as touching, may naturally occur less frequently as children grow older, potentially affecting entropy estimates.

Although not the primary focus of our study, we explored the associations between caregiving predictability and several covariates that have not yet been tested longitudinally in relation to unpredictability of maternal sensory signals. These insights may inform future research on caregiving predictability across developmental stages. Consistent with earlier findings, (Davis et al., 2017; Holmberg, et al., 2022a), our results showed no significant association between child sex and the unpredictability of maternal sensory signals. In turn, maternal age and educational level, reflecting socioeconomic status (SES), were negatively associated with the unpredictability of maternal sensory signals, suggesting that younger and less-educated mothers exhibited more unpredictable sensory signals. This aligns with prior research showing socioeconomic disadvantage and younger maternal age are risk factors for less favorable caregiving behaviors (e.g., Davis et al., 2019; Fall et al., 2015; Roubinov and Boyce, 2017). Moreover, while previous studies have demonstrated that cumulative maternal risk factors during infancy, such as low maternal self-regulation combined with high anxiety symptoms, or concurrent maternal postnatal depressive and anxiety symptoms, are associated with greater unpredictability in maternal sensory signals (Davis et al., 2017, Davis et al., 2019; Holmberg, et al., 2022a), our findings from a population-based birth cohort indicate that maternal depressive symptoms assessed longitudinally at the child's ages of 6 months, 2 years, and 5 years were not significantly associated with entropy rate. These results align with previous work from our research group, suggesting that, unlike maternal sensitivity, the unpredictability of maternal sensory signals may be less affected by maternal depressive and/or anxiety symptoms (Holmberg et al., 2022a). Nonetheless, as this is the first study to model entropy rate beyond toddlerhood, further research is needed to confirm and expand upon these findings.

The main findings of the present study indicated that the unpredictability of maternal sensory signals was associated with children's longitudinal EF profiles. Based on our previous study of the same sample (Karonen et al., 2025), three distinct EF profiles were identified: 1) “*Below average performers”* (14.2 %), 2) *“the Average performers with lower Spin the Pots (5 y) performance*” (56.0 %) and “*the Average performers with higher Spin the Pots (5 y) performance*” (29.8 %). The two latter groups were distinguished by their performance on a working memory task at the age of 5. Children in the average EF profile with better working memory task performance were exposed to less unpredictable maternal sensory signals compared to children in the two lower-performing EF profiles. The effect sizes for these comparisons were strong. Thus, more unpredictable maternal sensory signals were associated with poorer EF developmental profiles from infancy through early childhood, before school age. Our findings provide novel insight into the role of the unpredictability of maternal sensory signals as an important and unique aspect of early caregiving behavior, showing its specific associations with the early development of EFs, as well as other aspects of cognitive development and self-regulation capacity (Davis et al., 2017, Davis et al., 2019, Forest et al., 2025; Holmberg et al., 2022b; Hyysalo et al., 2024). In line with recent cross-species research (Baram et al., 2012, Davis et al., 2017, Granger et al., 2021), our results support the finding that patterns of sensory signals seem to play a crucial role not only in the development of sensory processing, but also in the development of higher-order neural circuits involved in EFs. Thus, our results suggest that, alongside other factors influencing child development, the unpredictability of maternal sensory signals plays a unique role in the development of EFs which undergo prolonged postnatal development, emphasizing an extended window of susceptibility to environmental influences beyond the infancy (Miguel et al., 2023).

This work contributes to the growing literature on proximal caregiving factors that shape early EF development. While prior research has emphasized the role of cognitive stimulation, scaffolding, and sensitive responsiveness in supporting EF skills (e.g., Bernier et al., 2010; Hammond et al., 2012)*,* unpredictability in maternal sensory signals offers a complementary perspective by focusing on the temporal structure of interactions rather than their content or emotional tone. Irregular patterns of sensory input may disrupt the child’s ability to form stable expectations and regulate attention, thereby influencing the development of EF components during a period when proximal caregiving serves as a primary source of environmental input relevant to cognitive development. This approach aligns with emerging models that emphasize the importance of consistency and predictability in early environments for the maturation of self-regulatory systems (Baram et al., 2012, Davis et al., 2017)*.*

The present study has several strengths, including its longitudinal design and a substantial number of participants, allowing for a comprehensive characterization of early EF development and changes in maternal entropy over time. We also used observational measures of caregiving predictability and child EF, minimizing the typical reported bias effects. However, there are also limitations in the present study. Although the missing data were primarily due to study design, this missingness may affect the generalizability of the findings to the broader population. Additionally, we used a limited set of EF measures that assessed only two specific dimensions, inhibition and working memory, rather than the more complex aspects of EF that are more directly linked to everyday behavior. While this variation could be seen as a limitation, it may also be considered a strength, as inhibitory control and working memory are thought to be the foundational EF core functions that emerge first. Future studies should examine how the unpredictability of maternal sensory signals relates to cognitive flexibility, which is one of the core components of EFs and typically emerges after working memory and inhibitory control in the hierarchical development of EF. Furthermore, the number and type of tasks varied between measurement points to account for the child’s developmental stage and, based on IRT analysis, some EF tasks demonstrated ceiling effects at age 5 (Yada et al., 2025), most notably the tablet-based working memory task Farmer, which limits the ability to determine which specific EF domain is most affected by early unpredictability. Thus, future studies should examine whether environmental unpredictability has domain-specific effects on EF development across different developmental stages. And finally, for future research, it would be important to investigate the stability and change of unpredictability in maternal caregiving also in high-risk populations, focusing particularly on longitudinal exposure to very high levels of proximal environmental unpredictability, to determine whether such exposure is associated with lower cognitive development. This would also help in identifying children who may benefit from early intervention efforts.

## 5 Conclusions

The unpredictability of maternal sensory signals during mother-child interactions is one of the pathways through which maternal care influences a child’s neurocognitive development, specifically EFs, from infancy to preschool years. In a truly longitudinal and novel setting, we found that lower unpredictability in maternal sensory signals was associated with better EF development in early childhood, suggesting that more predictable caregiving supports EF development. Notably, maternal sensory signals became more predictable as children grew older, emphasizing the need for further investigation into the stability of unpredictability in maternal sensory signals and the potential influence of children's cognitive and socioemotional development on the quality and dynamics of mother-child interactions. These unique findings enhance the current understanding of the nature and complexity of caregiving behavior's influence on a child’s self-regulation development. The unpredictability of maternal sensory signals within the caregiver-child dyad appears to be a critical environmental factor in early childhood, influencing the development of self-regulation and related cognitive processes.

## Data statement

The data necessary to reproduce the analyses presented here are not publicly accessible. The data protection legislation in Finland and ethics regulations of the FinnBrain Research do not permit open distribution of data, but data could be delivered by special requests (which includes formal collaboration and material transfer agreements). Investigators interested in research collaboration and obtaining access to the data are encouraged to contact FinnBrain board (finnbrain-board@lists.utu.fi). Contact information of the board members or FinnBrain Principal Investigators are listed on the project website: https://sites.utu.fi/finnbrain/en/contact/

The analytic code necessary to reproduce the analyses presented in this paper is not publicly accessible but is available from the corresponding author upon reasonable request.

The materials necessary to attempt to replicate the findings presented here are not publicly accessible.

The authors declare that the research was conducted in the absence of any commercial or financial relationships that could be construed as a potential conflict of interest.

## CRediT authorship contribution statement

**Fiia Takio**, Lead role**:** Conceptualization, Writing original draft, Visualization. Supporting role: data analysis. **Pilvi Peura**, Supporting role: Formal analysis, Methodology, Visualization. Supporting role: Writing review and editing. **Akie Yada**, Supporting role: Formal analysis, Methodology, Visualization. Supporting role: Writing review and editing. **Anniina Karonen**, Supporting role: Conceptualization, Investigation, Writing -review and editing. **Pauliina Juntunen**, Supporting role: Conceptualization, Methodology, Writing -review and editing **Eeva Holmberg**, Supporting role: Conceptualization, Investigation, Writing -review and editing. **Eeva Eskola**, Supporting role: Investigation, Methodology, Writing -review and editing. **Elisabeth Nordenswan** Supporting role: Investigation, Methodology, Writing -review and editing. **Kirby Deater-Deckard**, Supporting role: Methodology, Writing -review and editing. **Eeva-Leena Kataja**, Supporting role: Investigation, Methodology, Writing -review and editing. **Asko Tolvanen**, Supporting role: Methodology, Writing – review and editing. **Laura Perasto**: Supporting role: Methodology, Writing – review and editing. **Elina Mainela-Arnold**, Supporting role: Funding acquisition, Writing -review and editing. **Elysia Poggi Davis**, Supporting role: Methodology, Writing -review and editing. **Hasse Karlsson**, Lead role: Project administration, Supporting role: Funding acquisition, Writing -review and editing. **Linnea Karlsson**, Lead role: Project administration, Supporting role: Conceptualization, Funding acquisition, Writing -review and editing. **Saara Nolvi**, Lead role: Conceptualization, Funding acquisition, Supporting role: Writing -review and editing. **Riikka Korja**, Lead role: Conceptualization, Funding acquisition, Supervision, Supporting role: Writing -review and editing

## Funding

Fiia Takio: The 10.13039/501100003125Finnish Cultural Foundation.

Kirby Deater-Deckard: Fulbright-University of Turku Scholar Award, Fulbright Finland Foundation.

Saara Nolvi: Signe and Ane Gyllenberg Foundation, State Grants for Clinical Research, Emil Aaltonen Foundation, Finnish Cultural Foundation, Yrjö Jahnsson Foundation.

Riikka Korja: Research Council of Finland (308252, CoE, InterLearn, 346121), Finnish Cultural Foundation, Signe and Ane Gyllenberg Foundation, State Grants for Clinical Research.

Pauliina Juntunen: Signe and Ane Gyllenberg Foundation, The Finnish Cultural Foundation's Varsinais-Suomen Maakuntarahasto, The Olvi Foundation.

Anniina Karonen: Jenny and Antti Wihuri Foundation.

Elysia Poggi Davis: NIH/NIMH Conte Center P50 MH096889.

Linnea Karlsson: Research Council of Finland (#308176, #308589), Strategic Research Council established within the Research Council of Finland (#352649, #352655), Yrjö Jahnsson Foundation, Finnish Cultural Foundation, Signe and Ane Gyllenberg Foundation, Finnish State Grants for Clinical Research.

Eeva-Leena Kataja: 10.13039/501100002341Academy of Finland (#346790), Signe and Ane Gyllenberg Foundation Juho Vainio Foundation.

## Declaration of Competing Interest

The authors declare that they have no known competing financial interests or personal relationships that could have appeared to influence the work reported in this paper.

## Figures and Tables

**Table A.1:** Descriptive statistics for EF tasks and Entropy rate across age points

Measure	Age	N	Minimum	Maximum	M	SD
AnotB Score (theoretical maximum score 18)	8 months	369	0.00	16.00	4.83	3.80
Spin Score (theoretical max. score 16)	2.5 years	446	2.00	16.00	11.77	3.61
Snack Score (theoretical max. score 36)	2.5 years	394	0.00	36.00	27.49	8.53
Spin Score (theoretical max. score	5 years	528	6.00	20.00	11.36	3.40
DoGF Score (theoretical max. score 9)	5 years	541	0.00	9.00	6.04	3.12
Farmer Accuracy (theoretical max. score 1)	5 years	454	0.03	0.97	0.48	0.17
Arrows Accuracy in Incongruent Trials (1)	5 years	527	0.00	1.00	0.71	0.28
Pigs Accuracy (theoretical max. score 1)	5 years	512	0.33	1.00	0.96	0.08
Entropy rate	8 months	180	0.41	1.35	0.90	0.17
Entropy rate	2.5 years	378	0.30	1.27	0.78	0.14
Entropy rate	5 years	400	0.37	1.09	0.73	0.10

*Note*. AnotB = A not B, Spin = Spin the Pots, Snack = Snack Delay, DoGF = Delay of Gratification

**Table A.2:** Correlations for Main Variables

	1.	2.	3.	4.	5.	6.	7.	8.	9.	10.	11.	12.	13.	14.	15.	16.
1. Entropy 8 m	-															
2. Entropy 2.5 y	.30*	-														
3. Entropy 5 y	.30*	.23*	-													
4. EF: AnotB8m	.06	.10	.05	-												
5. EF: Snack2.5 y	.13	-.07	-.02	.02	-											
6. EF: Spin2.5 y	.05	-.06	-.06	.13	.23*	-										
7. EF: DoGF5y	.08	.01	-.11*	.03	.04	.08	-									
8. EF: Arrow5y	.12	.02	-.03	.07	.15*	.15*	.18*	-								
9. EF: Pigs5y	.00	.00	-.11*	.05	.26*	.13*	.07	.14*	-							
10. EF: Farmer5y	.02	.02	-.02	-.09	.11	-.03	.16*	.20*	.23*	-						
11. EF: Spin5y	-.02	-.17*	-.12*	-.09	.06	-.03	.03	.04	.17*	.04	-					
12. Maternal age	-.16*	-.13*	-.14*	-.07	-.08	-.04	-.09*	.07	.01	.03	.02	-				
13. Maternal education	-.09	-.16*	-.09	.05	.06	.10	.12*	.07	.11*	.02	-.04	.27*	-			
14. Financial satisfaction	-.20*	-.05	-.03	-.02	.07	.03	.05	.01	.05	.11*	-.05	.07*	.23*	-		
15. EPDS 6 m	-.16	.02	-.03	-.07	.09	-.17*	0.01	-.08	.07	-.03	-.05	-.01	-.01	-.05	-	
16. EPDS 2 y	.03	.00	-.08	-.05	-.09	-.02	-.08	-.05	-.06	.04	.12*	-.05	.07	-.14*	.59*	-
17. EPDS 6 y	.19	-.03	.01	-.06	-.05	.03	-.07	.03	.03	-.12	-.05	.05	-.06	-.12*	.46*	.55*

*Note.* AnotB = A not B, Snack = Snack Delay, Spin = Spin the Pots, DoGF = Delay of Gratification, EPDS = Edinburgh Postnatal Depression Scale. *p < .05.

## Data Availability

The data that has been used is confidential.
