# Supplementary material for: Unpredictable maternal signals and developmental profiles of child executive function from infancy to early childhood
Source: Dev Cogn Neurosci. 2026 Jan 9;78:101672. doi: 10.1016/j.dcn.2026.101672 (PMC12882702; doi:10.1016/j.dcn.2026.101672)
Supplement: Supplementary file 1 — Supplementary material [file mmc1.docx]

**Supplement**

**Child’s Executive function lab-based measurements**

At 8 months, infant’s ability to hold and update hiding location in mind (WM) and inhibit a prepotent response (IC) was measured with modified A-not-B -task (Nolvi et al., 2018; see also Diamond, 1985; Sun et al., 2009). In the task, the infant was seated in his/her mother’s lap in front of a table. An age appropriate toy was hidden in one of the two identical hiding locations placed 11 cm on either side of the midline of the child. Infants’ attention toward the task were supported by calling infant’s name. After infant gazed the midline, she/he was asked to identify the location of the toy. Stimuli were presented in a fixed pattern on three delay levels (0, 2, and 4 sec) each level consisting of 6 trials with changing position either right or left side (18 trials in total). The order of the side was counterbalanced. The direction of the infant’s reaching initiated within 8 sec was scored either as correct or as incorrect. After the series of trials in each delay, infants were allowed to continue to the next delay only if they scored correctly in 3 of the 6 trials. The procedure was video-recorded and coded by two coders. Inter-rater reliability was 81%. Higher score reflects better EF performance.

At 2.5 years, WM was measured with the Spin the Pots task (Hughes & Ensor, 2005) and IC was measured with modified Snack Delay task (Kochanska et al., 2000). In the Spin the Pots task, six distinct stickers were hidden under eight perceptually distinct boxes. All the boxes were placed on a rotating tray in front of the child. In each trial, the children were asked to choose one box with a sticker. After each trial, the boxes placed on the tray were covered by opaque scarf before rotating the tray 180o. The task was completed either when the child has found all six stickers or when the maximum number of 16 spins was reached. Total score was calculated by subtracting the number of unsuccessful attempts from the total number of trials the child went through. The total score was 16 at maximum and a higher score reflects better EF performance. In the modified Snack Delay task, the children were asked to place their hands on a mat with a picture for both hands while sitting at a table. The snacks (either candy or raisin) were placed under the opaque cup in front of the child. The child was told that she/he can eat the snack after the experimenter rang a bell. All together six trials with varying delays (range 10s to 60s) were conducted. In each trial, the experimenter picked up the bell without ringing it either once or twice before actually ringing the bell. From each trial, the range of scores were 0 to 4 (0 = child eats the snack before the bell was raised, 1 = child eats the snack after the bell was raised but before it was rung, 2 = child touches the cup or the bell before the bell was raised, 3 = child touches the cup or the bell after the bell was raised, 4 = child waits and takes the snack after the bell has rung). All children were given 2 extra points if they were able to keep their hands on the mat according to the instructions during the whole trial (Spinrad et al., 2007). The maximum score was 36 and a higher score reflects better IC performance.

At 5 years of age, EFs were measured with the Spin the Pots (WM) (Hughes and Ensor, 2005, see also Beck et al., 2011), the Delay of Gratification (IC) (Beck et al., 2011) and with three tasks from computerized Executive Function Touch (EF Touch) battery for preschoolers (the Farmer (WM), the Arrows (IC) and the Pig (IC)) (Willoughby, Blair, Wirth, Greenberg, et al., 2012; Willoughby et al., 2010, 2013; Willoughby et al., 2016). The EF Touch program is a Windows OS based program, where standard monitor displays a script to the interviewer while the child administers the task with touch screen tablet device. Mimicking the 2.5 years assessment, at the 5 years of age, the procedure of the Spin the Pots task was otherwise the same, but the number of stickers was 10 and of pots 12. The total score was 20 at maximum and a higher score reflects better EF performance. In the Delay of Gratification task, the child gets 9 trials to choose a smaller, immediate reward (0 points) or a larger, delayed reward (1 point) (Beck et al., 2011). Rewards were either stickers, edible treats, or 5-cent coins, in a standardized order. Based on child's choice, the child got the rewards either immediately or given to the child when leaving the visit. The maximum score was 9 with higher score depicting better IC. Visual Spatial Working Memory task (Farmer, EF Touch) included 36 items in total. In the task, children were presented with a 4 x 4 grid of squares that were referred to as farmer’s fields. At first, children watched the field where animal moved around in a sequence in the highlighted grinds (fields). After that, children were asked to touch the fields in the same order in which the animal highlighted the grids to help the farmer to find the animal. The difficulty level increased as the child was shown and asked to recall longer string of fields. The mean accuracy of responses for all items was analysed. Spatial Conflict task (Arrows, EF Touch) measuring IC and FX included 36 items in total. In the task, two buttons appeared on the left- and right-most side on the tablet screen. In the three different conditions, each consisting of 12 arrows, the direction of an arrow varied according to the spatial location of the buttons that the child was instructed to press (congruent, incongruent and mixed conditions). Each item was presented for 4,000 ms, and the accuracy and the reaction times for the responses were recorded. Responses that were faster than 400 ms were considered too fast to be plausible and were set to missing. If an item was omitted, the accuracy score was zero and reaction time was not recorded. In the analysis, mean accuracy for the number of incongruent items from incongruent and mixed conditions were used for the analysis. Animal Go/No-Go task (Pig, EF Touch) measuring IC included 40 items in total. In the task, children were instructed to touch a centrally located large green button on the touch screen on the tablet every time when they saw any animal on a screen (“go” item) except when the animal was a pig (“no-go” item). Each item was presented for 3,000 ms. The accuracy and the reaction time of each response were recorded. If an item was omitted, the accuracy score was zero and reaction time was not recorded. Only the accuracy of the responses was analysed. Item responses that were faster than 400 ms were considered too fast to be plausible and were treated as missing responses.

**Longitudinal modelling of EFs**

Following the analysis published in our previous research (Karonen et al., 2025), children’s longitudinal EF profiles were identified with latent profile analysis (LPA) with EF task estimates. The EF data set included 34.8%-56.3% missing data, which was missing at random based on A Lillte’s Missing Completely At Random test (Little, 1998), χ2 (207) = 238.46, p = .060. Missingness was handled in the latent Profile analysis (LPA) by using The Full Information Maximum Likelihood (FIML) method (Enders, 2010). Factor scores of EF tasks were calculated based on item response theory analysis to identify item-level difficulty and discrimination properties of EF tasks (for a detailed description of the IRT analysis for each task see Yada et al., 2025). In LPA, EF factor means were allowed to vary between the profiles. Various LPA solutions were estimated up to 5-profile solution. The appropriate profile solution was selected based on the Goodness-of-fit indexes with following criteria: Akaike Information Criterion (AIC), Bayesian information criterion (BIC; Schwartz, 1978), sample size-adjusted BIC (saBIC; Yang, 2006), and the Lo–Mendell–Rubin adjusted likelihood ratio test (LMR-A; Lo, Mendell, & Rubin, 2001) and Vuong–Lo–Mendell–Rubin (VLMR; Lo et al., 2001) likelihood ratio (LR) tests. For LLR and information criteria (BIC and aBIC) lower values indicate better model fits. A significant p value provided by the LRM and VLMR tests indicate a better fitting model than that with one class less. Further, the classification quality of profiles was considered with average latent class probabilities, in which values over .80 indicate a distinct classification (Geiser, 2012).

The longitudinal EF profiles were explored with LPA by estimating various LPA solutions until the highest possible profile-solution that could be identified with the data was reached (see Table 2 for the fit statistics with 1 through 5 profile solutions). Based on the VLMR, LMR-A, and BLRT a 2-profile solution fit the data better than a 1-profile solution (p < .001) and a 3-profile solution fit better than a 2-profile solution (p < .001). Based on VLMR and LMR-A (p = .050), however, a 4-profile solution did not fit better than a 3-profile solution. Based on these findings, the 3-profile solution was considered as the best-fitting solution (see Table S2).

Table S1. *Fit statistics for latent profile analysis for 1 through 5 class solutions*

| Classes | AIC | BIC | saBIC | Entropy | VLMR p value | LMR-A p value | | BLRT p value | | Class proportions | Average latent class probabilities |
| --- | --- | --- | --- | --- | --- | --- | --- | --- | --- | --- | --- |
| 1 | 9885.566 | 9961,109 | 9910,298 | 1 | N/A | | N/A | | N/A | 1.000 | 1.000 |
| 2 | 9718.062 | 9836.098 | 9756.707 | 0.692 | 0.0002 | | 0.0002 | | 0.0000 | .170/.830 | .875/ .920 |
| **3** | **9609.182** | **9769.71** | **9661.738** | **0.594** | **0.0002** | | **0.0002** | | **0.0000** | **.142/ .560/ .298** | **.871/ .755/.932** |
| 4 | 9554.516 | 9757.537 | 9620.984 | 0.585 | 0.0501 | | 0.0524 | | 0.0000 | .130/ .420/ .121/ .329 | .815/ .737/ .832/ .719 |
| 5 | 9494.403 | 9739.917 | 9574.783 | 0.563 | 0.0150 | | 0.0163 | | 0.0000 | .109/ .242/ .348/ .174/ .127 | .852/ .729/ .613/ .777/ .747 |

Note. SABIC = sample-size adjusted BIC; VLMR = Vuong-Lo-Mendell-Rubin likelihood ratio test; LMR-A = Lo-Mendell-Rubin adjusted likelihood ratio test; BLRT = bootstrapped likelihood ratio test. Age-relatedly residualized variables for Spin the Pots 2.5y, Spin the Pots 5y and Farmer 5y.

References

Beck, D. M., Schaefer, C., Pang, K. C., & Carlson, S. M. (2011). Executive function in preschool children: Test-retest reliability and criterion validity of the delay of gratification task. *Developmental Neuropsychology*, 36(4), 373–382. https://doi.org/10.1080/87565641.2011.555459

Diamond, A. (1985). Development of the ability to use recall to guide action, as indicated by infants' performance on AB. *Child Development*, 56(4), 868–883. https://doi.org/10.2307/1130463

Enders, C. K. (2010). *Applied missing data analysis*. New York, NY: Guilford Publications.

Geiser, C. (2012). *Data analysis with Mplus*. Guilford Press.

Hughes, C., & Ensor, R. (2005). Executive function and theory of mind in 2 year olds: A family affair? *Developmental Neuropsychology*, 28(2), 645–668. <https://doi.org/10.1207/s15326942dn2802_5>

Karonen, A., Juntunen, P., Yada, A., Takio, F., Nordenswan, E., Eskola, E., Deater-Deckard, K., Bridgett, D. J., Fernandes, M., Mainela-Arnold, E., Karlsson, H., Karlsson, L., Kataja, E.-L., Korja, R., & Nolvi, S. (2025). *Longitudinal Profiles of Executive Functioning from Infancy to Five Years of Age – A FinnBrain Birth Cohort Study*. https://doi.org/10.31234/osf.io/2w8kg_v1

Kochanska, G., Murray, K., & Harlan, E. T. (2000). Effortful control in early childhood: Continuity and change, antecedents, and implications for social development. *Developmental Psychology*, 36(2), 220–232. https://doi.org/10.1037/0012-1649.36.2.220

Little, R. J. A. (1988). A test of missing completely at random for multivariate data with missing values. *Journal of the American Statistical Association*, 83(404), 1198–1202. https://doi.org/10.1080/01621459.1988.10478722

Lo, Y., Mendell, N., & Rubin, D. B. (2001). Testing the number of components in a normal mixture. *Biometrika*, 88(3), 767–778. <https://doi.org/10.1093/biomet/88.3.767>

Nolvi, S., Raikkonen, K., Pesonen, A.-K., Heinonen, K., & Strandberg, T. E. (2018). Early executive functioning and later internalizing symptoms: A longitudinal study from infancy to middle childhood. Development and Psychopathology, 30(3), 1179–1192. <https://doi.org/10.1017/S0954579417001801>

Schwartz, G. (1978). Estimating the dimension of a model. *The Annals of Statistics*, 6(2), 461–464. https://doi.org/10.1214/aos/1176344136

Spinrad, T. L., Eisenberg, N., Smith, C. L., Kupfer, A., & Gaertner, B. M. (2007). Relations of maternal socialization and toddlers’ effortful control to children’s adjustment and social competence. Developmental Psychology, 43(5), 1170–1186. <https://doi.org/10.1037/0012-1649.43.5.1170>

Sun, J., Mohay, H., & O’Callaghan, M. (2009). A comparison of executive function in very preterm and term infants at 8 months corrected age. Early Human Development, 85(4), 225–230. <https://doi.org/10.1016/j.earlhumdev.2008.10.005>

Willoughby, M. T., Blair, C. B., Wirth, R. J., Greenberg, M. T., et al. (2012). The measurement of executive function at age 5: Psychometric properties of a new battery of tasks. *Psychological Assessment*, 24(1), 226–239. <https://doi.org/10.1037/a0025543>

Willoughby, M. T., Blair, C. B., Wirth, R. J., & Greenberg, M. (2010). The measurement of executive function at age 3 years: Psychometric properties and criterion validity of a new battery of tasks. Psychological Assessment, 22(2), 306–317. <https://doi.org/10.1037/A0018708>

Willoughby, M. T., Kuhn, L. J., Blair, C. B., Samek, A., & List, J. A. (2017). The test–retest reliability of the latent construct of executive function depends on whether tasks are represented as formative or reflective indicators. Child Neuropsychology, 23(7), 822–837. <https://doi.org/10.1080/09297049.2016.1205009>

Willoughby, M. T., Wirth, R. J., & Blair, C. B. (2011). Contributions of modern measurement theory to measuring executive function in early childhood: An empirical demonstration. Journal of Experimental Child Psychology, 108(3), 414–435. <https://doi.org/10.1016/j.jecp.2010.04.007>

Yada, A., Deater-Deckard, K., Takio, F., Nordenswan, E., Eskola, E., Karlsson, H., Karlsson, L., Korja, R., Nolvi, S., & Tolvanen, A. (2025). *Using Item Response Theory to address challenges with measuring executive function in infancy and early childhood*. <https://doi.org/10.31234/osf.io/v96ea_v1>

Yang, C.-C. (2006). Evaluating latent class analysis models in qualitative phenotype identification. *Computational Statistics & Data Analysis*, 50(4), 1090–1104. <https://doi.org/10.1016/j.csda.2004.11.004>
